# Clinical outcomes among patients with chronic kidney disease hospitalized with diabetic foot disorders: A nationwide retrospective study

**DOI:** 10.1002/edm2.277

**Published:** 2021-06-09

**Authors:** Michael Salim

**Affiliations:** ^1^ Department of Internal Medicine Mount Sinai Hospital Chicago IL USA

**Keywords:** amputation, chronic kidney disease, diabetic foot infection, diabetic foot ulcer, dialysis, end‐stage renal disease

## Abstract

**Introduction:**

Diabetic foot ulcerations or infections (DFUs/DFIs) are common complications of patients with diabetes. This study aimed to explore the impact of non‐dialysis and dialysis CKD on hospitalized patients with DFUs/DFIs.

**Methods:**

A retrospective cohort study was conducted using the National Inpatient Sample database for the years 2017 and 2018. Patients hospitalized for DFUs/DFIs were included in the study. The primary outcome was lower limb amputations. The secondary outcomes were inpatient mortality, sepsis, length of stay (LOS), total hospitalization charges (THC) and disposition.

**Results:**

A total of 121,815 hospitalizations were included (26.1% non‐dialysis CKD; 8.4% dialysis CKD). There was no significant difference in amputation rates between those on non‐dialysis CKD (adjusted odds ratio [aOR]: 0.96; 95% confidence interval [CI]: 0.87–1.06) and dialysis CKD (aOR: 1.04, [95% CI: 0.91–1.12]) when compared to non‐CKD group. Dialysis CKD group had increased odds of undergoing major amputation (aOR: 1.74, [95% CI: 1.32–2.29]), in‐hospital mortality (aOR: 3.77 [95% CI: 1.94–7.31]), sepsis (aOR: 1.83 [95% CI: 1.27–2.62]), longer LOS (adjusted mean difference [aMD]: 1.46 [95 CI: 1.12–1.80) and higher THC (adjusted mean difference [aMD]: $20,148 [95% CI: $15,968‐$24,327]). Non‐dialysis CKD group had increased odds of sepsis (aOR: 1.36 [95% CI: 1.02–1.82]), less likely to be discharged home (aOR: 0.87 [95% CI: 0.80–0.95]), longer LOS (aMD: 0.91 [95% CI 0.69–1.13]) and higher THC (aMD: $20,148 [95% CI: $15,968–$24,327]).

**Conclusion:**

Patients with CKD on dialysis had higher odds of undergoing major amputation. CKD increased the odds of in‐hospital morbidity and resource utilization, with the most significant is for those on dialysis.

## Graphical abstract

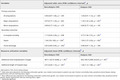

## 1 INTRODUCTION

Foot ulceration is one of the most common complications of patients with diabetes. The lifetime incidence has been predicted to be more than 19% in patients with diabetes.^1^ Approximately 58% of diabetic foot ulcers (DFU) would evolve into diabetic foot infection (DFI).^2^ DFUs cause a significant burden for the government and debility to the patients themselves. The total estimated cost for management of diabetic foot in the United States ranges from $9 to 13 billion annually in addition to diabetes care itself.^3^ Furthermore, up to one‐fifth of patients with moderate or severe DFU will lead to amputation and mortality.^1^, ^4^ Amputation will affect their ability to perform daily tasks, which in the end will negatively influence their quality of life.^5^ A study done by Wukich et al. showed that patients with diabetic foot perceived lower extremity amputation worse than death.^6^ Therefore, understanding the risk factors is an essential issue in order to perform early detection of foot complications.

Diabetes is also frequently complicated by kidney disease. Chronic kidney disease (CKD) is a known marker for the generalized vascular status of patients with diabetes. Overall, CKD is associated with an increased level of in‐hospital mortality, postoperative complications, length of stay and hospital costs.^7^, ^8^ Several studies have demonstrated an increase in morbidity and mortality in patients on dialysis who develop foot ulcers.^9^, ^10^, ^11^ However, studies on the influence of chronic kidney disease on diabetes‐related foot ulcer hospitalization have been inconsistent. Lee et al., in a case‐control study of 351 DFU subjects, demonstrated a significant relationship between low estimated glomerular filtration rate level and major amputation risk in patients diabetic foot.^12^ On the contrary, other investigations have shown no significant relationship between non‐dialysis CKD and DFUs/DFIs on hospital outcomes.^13^, ^14^

To our knowledge, the impact of non‐dialysis and dialysis CKD on patients with DFUs/DFIs has not been explicitly addressed among hospitalized patients at a national level. Therefore, this study aims to explore the impact of CKD on clinical outcomes and resource utilization.

## 2 MATERIALS AND METHODS

### 2.1 Study data and population

Data were sought from the 2016 and 2017 National Inpatient Sample (NIS) database. The NIS is a nationwide database of hospital inpatient stays across the United States, funded by the Agency for Healthcare Research and Quality (AHRQ). The NIS is part of the Healthcare Cost and Utilization Project (HCUP) and the largest publicly available national all‐payer inpatient healthcare database. The samples were collected from all US short‐term general and other specialty hospitals, excluding Federal hospitals, long‐term acute care hospitals and rehabilitation hospitals. Samples were stratified based on hospital census division, ownership, urban/rural location, teaching status and hospital beds. It collects data for more than 7 million unweighted hospital discharge records annually and approximately more than 35 million weighted hospitalizations annually. From 2017 to 2018, the NIS included over 71 million weighted discharge records from across 48 states. In 2017 and 2018, the NIS provides up to 40 diagnoses and 25 procedures for each hospitalization record. The NIS contains a large sample size; therefore, it is ideal for developing national and regional estimates of multiple conditions. The International Classification of Disease, 10^th^ Revision, Clinical Modification/Procedure Coding System (ICD‐10‐CM/PCS) coding system was used to report all medical diagnoses and inpatient procedures recorded in the NIS database. Detailed information is available at http://www.hcup‐us.ahrq.gov. ^15^

The NIS database was queried for patients 18 years and older who had a principal diagnosis of foot ulcers or foot infections and any diagnosis of diabetes mellitus using ICD‐10 codes. This cohort was further divided based on at least one secondary discharge diagnosis of non‐dialysis CKD and dialysis CKD. Institutional Review Board approval was not needed because the NIS database has completely removed possible patient identifiers, state level and hospital identifiers.^15^

### 2.2 Outcome measures

The primary outcome was any lower limb amputation. Lower limb amputation was further divided into major and minor amputation. Major amputation was defined as any amputation above the ankle joint. Minor amputation was defined as any amputation limited to the foot. The secondary outcomes included in‐hospital mortality, sepsis, disposition, length of stay and total hospitalization charges. Disposition was defined as either home discharge or all others (transfer to short‐term hospital, skilled nursing facility, intermediate care, home health care, against medical advice and died). ICD‐10‐CM/PCS codes were used to obtain lower limb amputation, major amputation, minor amputation and sepsis from the cohort (Table S1). The other outcome variables were available in the NIS database.

### 2.3 Statistical analysis

The data were analysed using STATA 16.0 (StataCorp). The NIS database represents a collection of a complex sampling design that includes weighting, clustering and stratification. All analyses were conducted using weighted samples based on guidelines outlined by HCUP NIS.^15^ Baseline characteristics of patients and hospitals were compared among patients presented with DFUs/DFIs based on CKD status using Pearson chi‐square for categorical variables and ANOVA for continuous variables. Potential confounders were identified based on previous literature,^10^, ^16^ and an initial univariate regression analysis was done with a cut‐off *p*‐value of .2. Adjusted confounders included the following: a) patient‐level: gender, race, median income based on patient's zip code, Charlson comorbidity index, peripheral arterial disease (PAD), obesity, hypertension and b) hospital level: bed size, teaching status, region. Multivariate regression analysis was used to adjust for possible confounders while calculating the primary and secondary outcomes. All *p*‐values were two‐sided, with.05 as the threshold for statistical significance.

## 3 RESULTS

### 3.1 Baseline characteristics

This retrospective, nationwide cohort study included 121,815 patients hospitalized for DFUs/DFIs in 2017 and 2018. Of these hospitalizations, there were 31,780 (26.1%) hospitalizations associated with non‐dialysis CKD and 10,285 (8.4%) hospitalizations associated with CKD on dialysis. Patients with non‐dialysis CKD and CKD on dialysis were older than those without CKD (mean age: 62.8 vs. 58.7 vs. 57.1, respectively, *p* < .001). The highest proportion among the three groups was dominated by Caucasians and males. The non‐dialysis CKD and CKD on dialysis groups also had higher Charlson comorbidities than those without CKD (score of ≥3: 99.1% vs. 99.6% vs. 33.6%, respectively, *p* < .001). The baseline characteristic of patients is presented in Table 1.

**TABLE 1:** Hospital and patient characteristics

Variables	Non‐CKD (*n* = 79,750)	Non‐dialysis CKD (*n* = 31,780)	Dialysis CKD (*n* = 10,285)	*p*
Age (years) (95% CI)	57.1 (56.9–57.3)	62.8 (62.5–63.2)	58.7 (58.1–59.3)	<.001
Female	31.0	31.0	34.1	.017
Race				
Caucasian	65.0	63.0	39.6	<.001
African American	14.6	19.0	32.3	
Hispanic	15.3	13.6	20.6	
Asian or Pacific Islander	0.9	1.33	2.3	
Native American	1.2	1.0	2.4	
Median income in patient's zip code^a^				
$1‐$45,999	37.3	34.1	41.0	<.001
$46,000–$58,999	28.4	28.3	26.0	
$59,000–$78,999	20.6	22.3	19.3	
$79,000 or more	13.6	15.3	13.7	
Insurance				
Medicare	37.3	34.1	41.0	<.001
Medicaid	28.4	28.3	26.0	
Private	20.6	22.3	19.3	
Uninsured	13.6	15.3	13.7	
Hospital bed size				
Small	23.2	22.3	18.0	<.001
Medium	32.0	31.1	32.0	
Large	44.7	46.6	50.0	
Hospital region				
Northeast	19.2	19.4	18.9	.006
Midwest	19.2	21.4	18.4	
South	42.5	40.2	42.6	
West	19.0	19.0	20.2	
Teaching hospital	63.8	66.4	73.6	<.001
Comorbidities				
Hypertension	65.3	2.4	1.3	<.001
Obesity	26.6	31.6	27.0	<.001
PAD	3.8	5.8	9.6	<.001
Charlson comorbidity index				
1	20.0	0.1	0.1	<.001
2	46.4	0.8	0.2	
3 or more	33.6	99.1	99.6	

Abbreviations: CI, confidence interval; CKD, chronic kidney disease; PAD, peripheral arterial disease.

^a^ For 2018.

### 3.2 Primary outcomes

Table 2 shows the results of the patient outcomes. In total, there were 27,610 estimated amputations (3420 major amputations and 24,190 estimated minor amputations) in the study population. Patients with CKD on dialysis had higher overall amputation and major amputation rates compared to those in non‐dialysis CKD and non‐CKD groups (25.1% vs. 23.3% vs. 22.1%, respectively, *p* = .010). However, the difference in minor amputation rates was not significant when compared between the groups (*p* = .664).

**TABLE 2:** Descriptive statistics of primary and secondary outcomes by extent of kidney disease

Variables	Non‐CKD (*n* = 79,750)	Non‐dialysis CKD (*n* = 31,780)	Dialysis CKD (*n* = 10,285)	*p*
Primary outcomes				
All amputations (%)	22.1	23.3	25.1	.010
Major amputations (%)	2.2	3.3	6.0	<.001
Minor amputations (%)	19.9	20.0	19.1	.664
Secondary outcomes				
In‐hospital mortality (%)	0.1	0.4	1.3	<.001
Sepsis (%)	1.4	2.0	2.3	.001
Home discharge (%)	50.9	37.6	37.0	<.001
Resource utilization variables				
Mean total hospitalization charges, mean (95% CI)	$48,555 ($47,499–$49,612)	$61,554 ($59,640–$63,467)	$77,737 ($73,774–$81,700)	<.000
Mean length of stay, mean (95% CI) (days)	5.63 (5.54–5.71)	7.01 (6.84–7.18)	7.67 (7.37–7.97)	<.000

Abbreviations: CI, confidence interval; CKD, chronic kidney disease.

Results of multivariate logistic regression analysis of the outcomes are shown in Table 3. After adjusting the confounders, there was no statistically significant difference in the adjusted odds ratio (aOR) of overall amputation rates between patients with non‐dialysis CKD and without CKD. However, patients with CKD on dialysis had an increase in odds of having major amputation when compared to patients without CKD (aOR 1.74 [CI: 1.32 – 2.29], *p* < .001).

**TABLE 3:** Associations between primary and secondary outcomes and extent of kidney disease

Variables	Adjusted odds ratio (95% confidence interval)^a^, *p*	
	Non‐dialysis CKD	Dialysis CKD
Primary outcomes		
All amputations	0.96 (0.87–1.06), *p* = .393	1.04 (0.91–1.12), *p* = .609
Major amputations	0.97 (0.77–1.23), *p* = .822	1.74 (1.32–2.29), *p* < .001*
Minor amputations	0.89 (0.77–1.02), *p* = .275	0.89 (0.77–1.02), *p* = .105
Secondary outcomes		
In‐hospital mortality	1.17 (0.60–2.29), *p* = .638	3.77 (1.94–7.31), *p* = .001*
Sepsis	1.36 (102–182), *p* = .036*	1.83 (1.27–2.62), *p* = .002*
Home discharge	0.87 (0.80–0.95), *p* = .002*	0.98 (0.87–1.10), *p* = .768

Resource utilization variables	Adjusted mean (95% confidence interval)^b^, *p*	
	Non‐dialysis CKD	Dialysis CKD
Additional total hospitalization charges	$6711 (4214–9208), *p* < .001*	$20,148 (15,968–24,327), *p* < .001*
Additional length of hospital stays	0.91 (0.69–1.13), *p* < .001*	1.46 (1.12–1.80), *p* < .001*

Abbreviations: CKD, chronic kidney disease.

^a^ Odds ratios were adjusted for the following confounders: gender, race, median income based on patient's zip code, Charlson comorbidity index, peripheral arterial disease, obesity, hypertension, bed size, teaching status, region.

^b^ Mean differences were adjusted for the following confounders: patient‐level: gender, race, median income based on patient's zip code, Charlson comorbidity index, peripheral arterial disease, obesity, hypertension, bed size, teaching status, region.

* Statistically significant.

### 3.3 Secondary outcomes

Table 2 shows the results of the patient outcomes. When compared with the patients without CKD, the non‐dialysis CKD and dialysis CKD groups had higher rate of in‐hospital mortality (0.1% vs 0.4% vs 1.3%, respectively; *p* < .001), sepsis (1.4% vs 2.0% vs 2.3%, respectively; *p* = .001), total hospitalization charges ($48,555 vs $61,554 vs $77,737, respectively; *p* < .001) and longer length of stay (5.63 days vs 7.01 days vs 7.67 days, respectively; *p* < .001). Moreover, patients with non‐dialysis CKD and dialysis CKD were less likely to be discharged to home compared to patients without CKD (37.6% vs 37.0% vs 50.9%, respectively; *p* < .001).

Results of multivariate logistic regression analysis of the outcomes are shown in Table 3. After adjustments, the non‐dialysis CKD group was associated with a significantly increased risk of sepsis (aOR: 1.36, 95% CI = 1.02–1.82; *p* = .036). In addition, the non‐dialysis CKD group was also significantly less likely to be discharged home (aOR: 0.87, 95% CI = 0.80–0.95; *p* = .002), had higher total hospitalization charges ($6711, 95% CI = $4214–$9208; *p* < .001) and had longer length of stay (0.91 days, 95% CI = 0.69–1.13; *p* < .001).

After adjusting for the potential confounders, the patients with CKD on dialysis were associated with a significantly increased risk of in‐hospital mortality (aOR: 3.77, 95% CI = 1.94–7.31; *p* = .001), sepsis (aOR: 1.83, 95% CI = 1.27–2.62; *p* = .002). Moreover, the CKD on dialysis group also significantly had higher total hospitalization charges ($20,148, 95% CI = $15,968–$24,327; *p* < .001) and longer length of stay (1.46 days, 95% CI = 1.12–1.80; *p* < .001).

## 4 DISCUSSION

The current study analysed 121,815 admissions from the NIS database to examine the in‐hospital outcomes of patients with CKD hospitalized for DFUs/DFIs. The results demonstrated that, while patients with non‐dialysis CKD did not have higher odds of amputation, patients with CKD on dialysis treatment had increased odds of major amputation. Compared to patients without CKD, patients with underlying CKD in both groups also had worse clinical outcomes and an increase in resource utilization, including length of stay and total hospitalization charges.

Several studies showed that CKD was associated with worse mortality and morbidity in patients with diabetic foot.^9^, ^10^, ^11^, ^12^ CKD promotes more severe peripheral vascular diseases by causing chronic inflammation, oxidative stress and inducing a prothrombotic state. The incidence of PAD was directly correlated with the stage of CKD.^17^ A significant association between CKD and major amputation was observed in a retrospective cohort study of 669 individuals with foot ulcers. Compared to CKD stage 3, those with CKD stage 4–5 and CKD on dialysis had a higher risk for major amputation (hazard ratios 9.5 and 15.0, respectively; *p* < .005).^18^ In addition, Sayiner et al. concluded that, in patients with DFUs, PAD tripled the odds of having major amputation.^19^ On the contrary, some studies showed no significant relationship between diabetic foot amputation and CKD or initiation of dialysis.^13^, ^14^, ^20^ Findings from the current study demonstrated that patients from non‐dialysis CKD and CKD on dialysis groups had higher overall amputations rates compared to patients without CKD. After adjusting PAD and other confounders, dialysis treatment still significantly increased the odds ratio of undergoing major amputation by 74%. One possible explanation is patients receiving dialysis treatment had worse kidney function compared to other groups. Furthermore, dialysis treatment itself might also decrease tissue oxygenation and blood flow of the foot. This effect was noticed to be more prominent in patients with diabetes than in patients without diabetes.^21^

CKD has consistently been linked to adverse cardiovascular and renal outcomes. This association was not only seen in advanced CKD but also stage 1 or 2 CKD.^22^ Poor clinical outcomes of chronic kidney disease patients in hospitalized patients have also been reported in the previous literature.^23^, ^24^, ^25^ Yoshihara et al. showed overall increased in‐hospital complications of patients with advanced CKD (aOR 3.34, 95% CI 3.09 – 3.60; *p* < .001) and patients on dialysis treatment (aOR 2.16, 95% CI 1.65 – 2.83; *p* < .001) when compared to patients with non‐advanced CKD.^23^ Dialysis was also reported to increase the risk of inpatient mortality ten to twenty times and overall complication in patients undergoing total hip and knee arthroplasty.^24^ Furthermore, Minakata et al. demonstrated that the risk of infection post‐coronary artery bypass was doubled even in stage 2 CKD.^25^ These studies are consistent with the results from the present study. The odds of in‐hospital mortality were increased by almost fourfold in the dialysis group. A higher incidence of sepsis was also noticed in CKD patients, with the highest incidence observed in those on dialysis.

The present study also demonstrated the financial burden of DFUs/DFIs in the non‐dialysis CKD and dialysis CKD population. Both groups in this study showed an increase in resource utilization, including total hospitalization charges and hospital stays. Some of the differences in expense might be accounted for additional inpatient dialysis and nephrology consult. However, patients with non‐dialysis CKD also faced higher hospitalization charges and more extended hospital stays. Worse clinical outcomes, which were evident in both groups, could have also translated into higher hospitalization costs. These findings were consistent with previous studies investigating the impact of patients with non‐dialysis CKD and CKD on dialysis on other medical conditions, including acute pancreatitis, post‐prostatectomy and heart failure.^8^, ^26^, ^27^

This study has some important limitations. Firstly, the NIS database uses ICD‐10 codes to characterize diagnoses, procedures and hospitalization events. The database does not provide laboratory or imaging parameters and degrees or extent of DFUs/DFIs. Therefore, there is a possibility of misclassification of the diagnoses. Secondly, CKD might be under‐reported in the NIS database. ICD‐10 codes show a high accuracy for diabetic foot complications and high specificity and low sensitivity for CKD.^28^, ^29^ Consequently, some CKD patients were included in the non‐CKD group. It means that the misclassification introduced would cause the statistic results more significant. Lastly, there is a risk of residual or unmeasured confounders in the retrospective analysis.

## 5 CONCLUSION

The present study demonstrated that the CKD population, particularly the dialysis CKD group, is posed to higher amputation rates, worse clinical outcomes and more enormous economic impacts on patients with DFUs/DFIs. The results from this study highlight the need for more research on ways to prevent diabetic foot complications in this high‐risk population.

## CONFLICTS OF INTERESTS

The author reports no conflicts of interest in this work.

## AUTHOR CONTRIBUTIONS

MS contributed in the conceptualization, data curation, formal analysis, methodology, software, validation, visualization, draft preparation, review and editing.

## Supporting information

Table S1

## Data Availability

All data generated or analysed during this study are publicly available from the data repositories HCUP database. The database is available online at http://www.hcup‐us.ahrq.gov.^15^

## References

[1] Armstrong DG , Boulton AJM , Bus SA . Diabetic foot ulcers and their recurrence. N Engl J Med. 2017;376(24):2367‐2375. 10.1056/NEJMra1615439. 28614678

[2] Prompers L , Huijberts M , Apelqvist J , et al. High prevalence of ischaemia, infection and serious comorbidity in patients with diabetic foot disease in Europe. Baseline results from the Eurodiale study. Diabetologia. 2007;50(1):18‐25. 10.1007/s00125-006-0491-1. 17093942

[3] Rice JB , Desai U , Cummings AKG , Birnbaum HG , Skornicki M , Parsons NB . Burden of diabetic foot ulcers for medicare and private insurers. Diabetes Care. 2014;37(3):651‐658. 10.2337/dc13-2176. 24186882

[4] Armstrong DG , Swerdlow MA , Armstrong AA , Conte MS , Padula WV , Bus SA . Five year mortality and direct costs of care for people with diabetic foot complications are comparable to cancer. J Foot Ankle Res. 2020;13(1):16. 10.1186/s13047-020-00383-2. 32209136 PMC7092527

[5] Raspovic KM , Ahn J , La Fontaine J , Lavery LA , Wukich DK . End‐stage renal disease negatively affects physical quality of life in patients with diabetic foot complications. Int J Low Extrem Wounds. 2017;16(2):135‐142. 10.1177/1534734617707081. 28682731

[6] Wukich DK , Raspovic KM , Suder NC . Patients with diabetic foot disease fear major lower‐extremity amputation more than death. Foot Ankle Spec. 2018;11(1):17‐21. 10.1177/1938640017694722. 28817962

[7] Hsiue PP , Seo LJ , Sanaiha Y , Chen CJ , Khoshbin A , Stavrakis AI . Effect of kidney disease on hemiarthroplasty outcomes after femoral neck fractures. J Orthop Trauma. 2019;33(11):583‐589. 10.1097/BOT.0000000000001576. 31343596

[8] Lemor A , Hernandez GA , Lee S , et al. Impact of end stage renal disease on in‐hospital outcomes of patients with systolic and diastolic heart failure (insights from the Nationwide Inpatient Sample 2010 to 2014). Int J Cardiol. 2018;266:174‐179. 10.1016/j.ijcard.2018.02.117. 29887443

[9] Miyajima S , Shirai A , Yamamoto S , Okada N , Matsushita T . Risk factors for major limb amputations in diabetic foot gangrene patients. Diabetes Res Clin Pract. 2006;71(3):272‐279. 10.1016/j.diabres.2005.07.005. 16139385

[10] O’Hare AM , Bacchetti P , Segal M , Hsu C , Johansen KL . Dialysis morbidity and mortality study waves. Factors associated with future amputation among patients undergoing hemodialysis: results from the Dialysis Morbidity and Mortality Study Waves 3 and 4. Am J Kidney Dis Off J Natl Kidney Found. 2003;41(1):162‐170. 10.1053/ajkd.2003.50000. 12500233

[11] Orimoto Y , Ohta T , Ishibashi H , et al. The prognosis of patients on hemodialysis with foot lesions. J Vasc Surg. 2013;58(5):1291‐1299. 10.1016/j.jvs.2013.05.027. 23810259

[12] Lee JH , Yoon JS , Lee HW , et al. Risk factors affecting amputation in diabetic foot. Yeungnam Univ J Med. 2020;37(4):314‐320. 10.12701/yujm.2020.00129. 32370489 PMC7606965

[13] Ugwu E , Adeleye O , Gezawa I , Okpe I , Enamino M , Ezeani I . Predictors of lower extremity amputation in patients with diabetic foot ulcer: findings from MEDFUN, a multi‐center observational study. J Foot Ankle Res. 2019;12:34. 10.1186/s13047-019-0345-y. 31223342 PMC6570910

[14] Shatnawi NJ , Al‐Zoubi NA , Hawamdeh HM , Khader YS , Garaibeh K , Heis HA . Predictors of major lower limb amputation in type 2 diabetic patients referred for hospital care with diabetic foot syndrome. Diabetes Metab Syndr Obes Targets Ther. 2018;11:313‐319. 10.2147/DMSO.S165967. PMC6018853 29950877

[15] HCUP‐US Home Page. Accessed April 9, 2021. https://www.hcup‐us.ahrq.gov.

[16] Harris CM , Abougergi MS , Wright S . Clinical outcomes among morbidly obese patients hospitalized with diabetic foot complications. Clin Obes. 2019;9(1):e12285. 10.1111/cob.12285. 30288938

[17] Chen J , Mohler ER , Xie D , et al. Risk factors for peripheral arterial disease among patients with chronic kidney disease. Am J Cardiol. 2012;110(1):136‐141. 10.1016/j.amjcard.2012.02.061. 22465315 PMC3586781

[18] Otte J , van Netten JJ , Woittiez A‐JJ . The association of chronic kidney disease and dialysis treatment with foot ulceration and major amputation. J Vasc Surg. 2015;62(2):406‐411. 10.1016/j.jvs.2015.02.051. 25937604

[19] Sayiner ZA , Can FI , Akarsu E . Patients’ clinical charecteristics and predictors for diabetic foot amputation. Prim Care Diabetes. 2019;13(3):247‐251. 10.1016/j.pcd.2018.12.002. 30600172

[20] Lavery LA , Lavery DC , Hunt NA , Fontaine JL , Lavery RD . Does the start of dialysis initiate a period of increased risk of ulceration or amputation? J Am Podiatr Med Assoc. 2018;108(1):1‐5. 10.7547/16-056. 29547031

[21] Game FL , Selby NM , McIntyre CW . Chronic kidney disease and the foot in diabetes ‐ Is inflammation the missing link. Nephron Clin Pract. 2013;123(1–2):36‐40. 10.1159/000351813. 23752138

[22] Brantsma AH , Bakker SJL , Hillege HL , et al. Cardiovascular and renal outcome in subjects with K/DOQI stage 1–3 chronic kidney disease: the importance of urinary albumin excretion. Nephrol Dial Transplant. 2008;23(12):3851‐3858. 10.1093/ndt/gfn356. 18641082

[23] Yoshihara H , Yoneoka D . In‐hospital outcomes of patients with advanced chronic kidney disease, dialysis, and kidney transplant undergoing spinal fusion: analysis of a nationwide database. Clin Spine Surg. 2018;31(9):400‐405. 10.1097/BSD.0000000000000692. 30024446

[24] Ponnusamy KE , Jain A , Thakkar SC , Sterling RS , Skolasky RL , Khanuja HS . Inpatient mortality and morbidity for dialysis‐dependent patients undergoing primary total hip or knee arthroplasty. J Bone Joint Surg Am. 2015;97(16):1326‐1332. 10.2106/JBJS.N.01301. 26290083

[25] Minakata K , Bando K , Tanaka S , et al. Preoperative chronic kidney disease as a strong predictor of postoperative infection and mortality after coronary artery bypass grafting. Circ J Off J Jpn Circ Soc. 2014;78(9):2225‐2231. 10.1253/circj.cj-14-0328. 25070504

[26] Kroner PT , Mareth K , Raimondo M , et al. Acute pancreatitis in advanced chronic kidney disease and kidney transplant recipients: results of a US nationwide analysis. Mayo Clin Proc Innov Qual Outcomes. 2019;3(2):160‐168. 10.1016/j.mayocpiqo.2019.03.006. 31193877 PMC6543454

[27] Ning C , Hu X , Liu F , et al. Post‐surgical outcomes of patients with chronic kidney disease and end stage renal disease undergoing radical prostatectomy: 10‐year results from the US National Inpatient Sample. BMC Nephrol. 2019;20(1):278. 10.1186/s12882-019-1455-2. 31337353 PMC6651956

[28] Lowe JR , Raugi G , Reiber GE , Whitney JD . Changes in classifications of chronic lower‐limb wound codes in patients with diabetes: ICD‐9‐CM versus ICD‐10‐CM. Adv Skin Wound Care. 2015;28(2):84‐92. 10.1097/01.ASW.0000459576.85574.3f. 25608014

[29] Fleet JL , Dixon SN , Shariff SZ , et al. Detecting chronic kidney disease in population‐based administrative databases using an algorithm of hospital encounter and physician claim codes. BMC Nephrol. 2013;14(1):81. 10.1186/1471-2369-14-81. 23560464 PMC3637099

