# Supplementary material for: Clinical outcomes among patients with chronic kidney disease hospitalized with diabetic foot disorders: A nationwide retrospective study
Source: Endocrinol Diabetes Metab. 2021 Jun 9;4(3):e00277. doi: 10.1002/edm2.277 (PMC8279616; doi:10.1002/edm2.277)
Supplement: Supplementary file 1 — Table S1 [file EDM2-4-e00277-s001.docx]

**Table S1**

| **Variables** | **International Classification of Disease, 10^th^ Revision, Clinical Modification/Procedure Coding System (ICD-10-CM/PCS)** |
| --- | --- |
| Foot ulcer | L97.30x, L97.31x, L97.32x, L97.40x, L97.41x, L97.42x, L97.50x, L97.51x, L97.52x, L03.03x, L03.04x, M86.07x, M86.17x, M86.27x, M86.37x, M86.47x, M86.57x, M86.67x, M86.8X7, L02.6x, M65.07x, M71.07x |
| Diabetes Mellitus | E10x, E11x |
| Non-dialysis Chronic Kidney Disease | I12.9, N18.1, N18.2, N18.3, N18.4, N18.5, N18.9, I13.0, I13.10 |
| Dialysis Chronic Kidney Disease | N18.6, I12.0, I13.11, I13.2  5A1D00Z, 5A1D60Z, 3E1M39Z |
| Acute Kidney Failure | N17x |
| Major amputation | 0Y6H0Z3, 0Y6J0Z3, 0Y6H0Z1, 0Y6H0Z2, 0Y6H0Z3, 0Y6J0Z1, 0Y6J0Z2, 0Y6J0Z3, 0Y6F0ZZ, 0Y6G0ZZ, 0Y6C0Z1, 0Y6C0Z2, 0Y6C0Z3, 0Y6D0Z1, 0Y6D0Z2, 0Y6D0Z3, 0Y670ZZ, 0Y680ZZ, 0Y620ZZ, 0Y630ZZ, 0Y640ZZ |
| Minor amputation | 0Y6M0Z0, 0Y6N0Z0, 0Y6P0Zx,, 0Y6Q0Zx, 0Y6R0Zx, 0Y6S0Zx, 0Y6U0Zx, "0Y6V0Zx, 0Y6W0Zx, 0Y6X0Zx, 0Y6Y0Zx, 0Y6M0Zx, 0Y6N0Zx, 0Y6T0Zx |
| Sepsis | A41x, R65.2x, A40x, A02.1, A32.7, A54.86, B37.7, T81.12 |
| Peripheral arterial disease | I70.2x, I70.8, I70.9x, I74.4, I77.89, I77.9 |
| Hypertension | I10 |
| Obesity | E66x, Z68.3x, Z68.4x |
